# Mapping the selection, availability, price and affordability of essential medicines for mental health conditions at a global level

**DOI:** 10.1017/S2045796022000087

**Published:** 2022-04-19

**Authors:** B. Todesco, G. Ostuzzi, C. Barbui

**Affiliations:** 1 WHO Collaborating Centre for Research and Training in Mental Health and Service Evaluation; Department of Neuroscience, Biomedicine and Movement Sciences; Section of Psychiatry, University of Verona, Verona, Italy; 2 Cochrane Global Mental Health, University of Verona, Verona, Italy

**Keywords:** Mental health, essential medicines, availability, affordability, prices

## Abstract

**Aims:**

To provide a cross-country analysis of selection, availability, prices and affordability of essential medicines for mental health conditions, aiming to identify areas for improvement.

**Methods:**

We used the World Health Organization (WHO) online repository of national essential medicines lists (EMLs) to extract information on the inclusion of essential psychotropic medicines within each country's EML. Data on psychotropic medicine availability, price and affordability were obtained from the Health Action International global database. Additional information on country availability, prices and affordability of essential medicines for mental disorders was identified by searching, up to January 2021, PubMed/Medline, CINAHIL, Scopus and the WHO Regional Databases. We summarised and compared the indicators across lowest-price generic and originator brand medicines in the public and private sectors, and by country income groups.

**Results:**

A total of 112 national EMLs were analysed, and data on psychotropic medicine availability, price and affordability were obtained from 87 surveys. While some WHO essential psychotropic medicines, such as chlorpromazine, haloperidol, amitriptyline, carbamazepine and diazepam, were selected by most national lists, irrespective of the country income level, other essential medicines, such as risperidone or clozapine, were included by most national lists in high-income countries, but only by a minority of lists in low-income countries. Up to 40% of low-income countries did not include medicines that have been in the WHO list for decades, such as long-acting fluphenazine, lithium carbonate and clomipramine. The availability of generic and originator psychotropic medicines in the public sector was below 50% for all medicines, with low-income countries showing rates lower than the overall average. Analysis of price data revealed that procurement prices were lower than patient prices in the public sector, and medicines in the private sector were associated with the highest prices. In low-income countries, the average patient price for amitriptyline and fluoxetine was three times the international unit reference price, while the average patient price for diazepam was ten times the international unit reference price. Affordability was higher in the public than the private sector, and in high-income than low-income countries.

**Conclusion:**

Access to medicines for mental health conditions is an ongoing challenge for health systems worldwide, and no countries can claim to be fully aligned with the general principle of providing full access to essential psychotropic medicines. Low availability and high costs are major barriers to the use of and adherence to essential psychotropic medicines, particularly in low-and middle-income countries.

## Introduction

Mental health conditions are a global leading cause of illness and disability (Wang *et al*., 2007), accounting for more than a quarter of years lived with disability and for about 7.4% of disease burden worldwide (Whiteford *et al*., 2013). Facing such an important burden represents a global health priority and a major challenge for national health systems, particularly in low resource settings (Wang *et al*., 2007).

Profound disparities in the provision of adequate mental healthcare have been reported between rich and developing countries. At the heart of these inequities lies the difficulty to have access to evidence-based treatment and care (Patel and Prince, 2010). The extent of this ‘treatment gap’ for mental health conditions is so huge that more than 75% of people in low- and middle-income countries does not receive any mental healthcare, and do not access basic psychotropic medicines (Demyttenaere *et al*., 2004). Access to basic psychotropic medicines is a first, crucial step in improving global access to mental healthcare and reducing the treatment gap (Barbui *et al*., 2016).

The concept of ‘essential medicines’ was developed to identify a selection of medicines whose relevance for public health, efficacy, safety profile and cost effectiveness makes them ‘more important’ than others (Wirtz *et al*., 2017; Cappello *et al*., 2020). Therefore, essential medicines should be available for free or at affordable prices to those in need (Gray *et al*., 2015). Since 1977 an essential medicines list (EML) has been drawn up by the WHO to provide countries with a guide in their own choices for national EMLs (Laing *et al*., 2003). The inclusion of a particular medicine in national EMLs has been shown to result in higher availability compared to non-essential medicines, particularly in the public sector and in low- and middle-income countries (Bazargani *et al*., 2014). However, availability differences were observed between medicine for acute and chronic diseases in low- and middle-income countries, with generic medicines for chronic conditions being significantly less available in both public and private systems compared to medicines for acute conditions (Cameron *et al*., 2011; Beran *et al*., 2014). More generally, essential medicines availability has been shown to be still suboptimal in many countries (Richards, 2006; Mahmic-Kaknjo *et al*., 2018).

Growing evidence evaluating access to essential medicine for the chronic non-communicable disease has been published in recent years. Medicine availability, prices and affordability across a large range of countries were investigated for several medicine categories, particularly antiepileptic (Cameron *et al*., 2012), diabetes (Babar *et al*., 2019) and cardiovascular medicines (Husain *et al*., 2020). To our knowledge, no such study has ever been conducted in the field of mental health. Hence, we sought to conduct an analysis of essential psychotropic medicine selection, availability, price and affordability across a wide range of low, middle and high-income countries, aiming to better understand the extent to which essential medicines for mental health conditions are accessible to those in need at a global level.

## Method

### Selection of WHO essential psychotropic medicines

The 21st WHO EML was accessed to identify essential medicines for mental health conditions. The WHO EML consists of a core list of medicines considered essential for basic health-care needs, and a complementary list of additional essential medicines for which specialist diagnostic and/or monitoring facilities are required (WHO, 2019). For each mental health conditions, the listed essential medicines were extracted, recording whether they were included as individual medicines or as representatives of a specific pharmacological class. In the latter case, the WHO EML includes an accompanying ‘square box’ symbol (Cappello *et al*., 2020). For each medicine, the formulation recommended by WHO was also recorded.

National EMLs were accessed from the WHO repository of the National Medicines List/Formulary/Standard Treatment Guidelines. From each national EML, we recorded which WHO essential medicines for mental health conditions were included (WHO, 2021g). As a check of data accuracy, official country government webpages were additionally screened to check for the presence of updated national EML versions not stored in the WHO repository. When more than one national EML was found for the same country, the most recent was considered.

### Availability, prices and affordability of WHO essential psychotropic medicines

Data on country availability, price and affordability of WHO essential medicines for mental health conditions were extracted from the Health Action International (HAI) global database, a repository of results of national and sub-national medicine access surveys carried out using the WHO/HAI standardised methodology (HAI, 2021). All country surveys reporting data on any WHO essential psychotropic medicines were considered in the analysis.

From each survey, data on medicine availably in the public and private sectors were extracted for the originator brand and lowest price generic. Availability was expressed as the mean percentage of facilities in which the medicine was found on the day of data collection (HAI, 2021).

Three price indicators were extracted for the lowest price generic: the public-sector procurement price, the patient purchase price in the public sector, and the patient purchase price in the private sector. Public-sector procurement price represents the cost that government and other purchasers must pay to assure medicine supply. Patient price in the public sector is the medicine cost for patients in government, municipality or other local authority health facilities, including clinics and hospitals, health centres and pharmacies. Patient price in the private sector is the price that patients need to pay in retail pharmacies and private clinics or hospitals (HAI, 2021).

Data on medicine affordability in the public and private sectors were extracted for the originator brand and lowest price generic. Affordability was expressed as the number of day's wages that the lowest-paid unskilled worker would need to purchase a defined course of treatment for a specific mental health condition (HAI, 2021).

Additional data on country availability, prices and affordability of essential medicines for mental health conditions were identified by searching, from 2010 onwards and without language restrictions, the following databases: PubMed/Medline, CINAHIL, Scopus, African Index Medicus, Index Medicus for the Eastern Mediterranean Region, Index Medicus for the South-East Asian Region, Latin American and Caribbean Health Sciences Literature and Western Pacific Region Index Medicus. The following search terms were used: (essential[Title/Abstract]) AND (medicine*[Title/Abstract]) AND ((availability[Title/Abstract]) OR (affordability[Title/Abstract]) OR (selection[Title/Abstract])) AND (2010:2021[pdat]) AND (2010:2021[pdat]). Surveys reporting national or province or local data on availability, price and affordability of WHO essential medicines for mental health conditions were included only if the WHO/HAI methodology was followed. Two review authors independently screened titles and abstracts for inclusion. Articles rated as possible candidates by either of the two reviewers were retrieved. Working independently and in duplicate, the two review authors inspected the full texts for inclusion. Discrepancies between reviewers were resolved by consensus or through discussion with the research team. The PRISMA guidance for the conduct and reporting of systematic reviews was followed (Moher *et al*., 2009). For each included survey, we extracted information on the year and country of publication. Availability, price and affordability data were extracted by two researchers in duplicate, following the WHO/HAI methodology.

### Data analysis

An excel spreadsheet was populated with selection, availability, price and affordability data extracted from the WHO/HAI repository and from the additional surveys identified by the literature search. When more than one survey was available for a country, the most recent was chosen. If multiple sub-regional surveys were available, they were counted separately in the analysis.

For selection data, the proportion of countries that included WHO essential psychotropic medicines in national EMLs was calculated. Selection data were presented by income level and WHO Region. For availability data, when only median percentages were reported, mean values were estimated using the methods of McGrath and colleagues (McGrath *et al*., 2020). Overall mean values were then calculated, for each essential psychotropic medicine, grouping countries by income level and WHO Region.

In order to enable cross country price comparisons, price data were expressed as median price ratios (MPRs). MPR was calculated as the median local unit price divided by the international unit reference price in local currency, using the exchange rate on the first day of data collection. The Management Sciences for Health (MSH) International Drug Price Indicator Guide was used as a reference price (MSH, 2021). To allow comparison of MPR from different years, all MPRs were converted into a base-year (namely 2010), following the methodology defined in the WHO/HAI manual (HAI, 2021). The different sources of the exchange rate used in the surveys were standardised against International Monetary Fund rates, and the Consumer Price Index was used to adjust prices for inflation/deflation. Patient prices were also adjusted for purchasing power parity, to account for differences in purchasing power of individual currencies. Adjusted mean MPRs were presented, for each essential psychotropic medicine, grouping countries by income level and WHO Region. For affordability data, the overall median number of days of work to purchase a defined course of treatment was presented, for each essential psychotropic medicine, grouping countries by income level and WHO Region. The World Bank criteria were used to group countries into low, lower-middle, upper-middle and high-income. Specifically, low-income economies were defined as those with a Gross Nation Income (GNI) per capita of $1045 or less in 2020, lower-middle-income economies as those with a GNI per capita between $1046 and $4,095, upper-middle-income economies are those with a GNI per capita between $4096 and $ 12 695 and high-income economies are those with a GNI per capita of $ 12 696 or more (World Bank, 2021).

## Results

A total of 112 NEMLs were extracted from the WHO repository of National Medicines List/Formulary/Standard Treatment Guidelines, and the inclusion of WHO essential psychotropic medicines was recorded. Medicine availability, price and affordability data were obtained from 87 surveys stored in the WHO/HAI repository. Additional availability, price and affordability information were obtained from 19 studies that used the WHO/HAI methodology (PRISMA flow-diagram and list of included/excluded studies reported in online supplement). A tabular list of included countries with a source of data and type of data is reported in the online supplement.

The proportion of national EMLs including WHO essential psychotropic medicines is presented in Table 1. While some WHO essential psychotropic medicines, such as chlorpromazine, haloperidol, amitriptyline, carbamazepine and diazepam, were selected by most national EMLs, irrespective of the country income level, there were essential medicines, such as risperidone or clozapine, included by most national EMLs in high-income countries, but only by a minority of lists in low- and middle-income countries. Up to 40% of low-income countries, in addition, did not include medicines that have been in the WHO list for decades, such as long-acting fluphenazine, lithium carbonate and clomipramine (Table 1).

**Table 1.** Proportion of national essential medicine lists including WHO essential psychotropic medicines, by medicine and country income level

Mental health condition	Essential psychotropic medicine		Income level		
		Low (*N* = 20) (%)	Lower middle (*N* = 41) (%)	Upper middle (*N* = 37) (%)	High (*N* = 14) (%)
Psychotic disorders	Chlorpromazine	100	90	97	100
	LAI fluphenazine	60	78	76	79
	Haloperidol	85	90	100	93
	Risperidone	35	61	14	100
	Clozapine	25	29	62	71
Depressive disorders	Amitriptyline	90	82	100	100
	Fluoxetine	65	76	95	100
Mood disorders	Carbamazepine	90	88	100	100
	Lithium carbonate	60	59	87	93
	Valproic acid	80	82	89	100
Anxiety	Diazepam	95	98	100	100
OCD	Clomipramine	60	49	57	57

OCD, Obsessive-Compulsive Disorder; LAI, long-acting injectable.

**Fig. 1.**
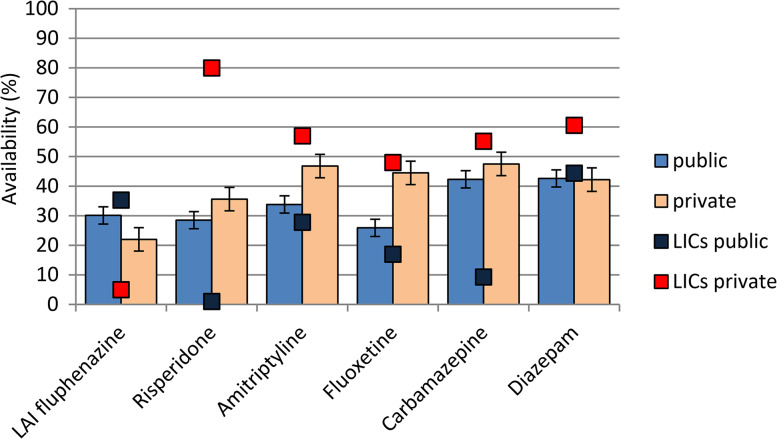
Public and private sector availability of lowest-price generic essential psychotropic medicines. Average (%) availability data for all countries surveyed (histograms) and for low-income countries (boxes) are reported.

Availability of lowest-price generics in the public sector ranged between 26% for fluoxetine and 43% for diazepam, with lower availability in low-income countries for most medicines (Fig. 1). In the private sector, essential psychotropic medicines were generally more available, with the exception of long-acting fluphenazine (Fig. 1). Originator brand availability in the public sector was low, ranging from 1.7% for risperidone to 17.7% for carbamazepine (online supplement), while in the private sector it ranged from 21.5% for fluphenazine to 52.3% for carbamazepine (online supplement). Lowest-price generic and originator brand availability by WHO Region did not show any clear pattern (online supplement).

**Fig. 2.**
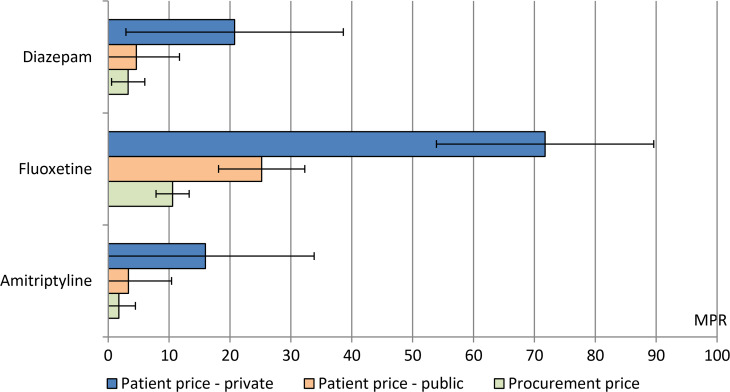
Procurement and patient prices of lowest-price generic psychotropic medicines in the public and private sector, expressed as median price ratios (MPRs).

Analysis of price data, expressed as MPRs, for lowest-price generic psychotropic medicines are presented in Table 2. In general, procurement prices were lower than patient prices in the public sector, and medicines in the private sector were associated with the highest MPRs (Table 2). In the public sector of low-income countries, the average patient price for amitriptyline and fluoxetine was three times the international unit reference price, while the average patient price for diazepam was ten times the international unit reference price (Table 2). Price analysis by WHO Region did not show any clear pattern (online supplement).

**Table 2.** Procurement and patient prices of lowest-price generic psychotropic medicines in the public and private sector by income level, expressed as median price ratios

Psychotropic medicine	Price	Sector	Income level			
			Low MPR (*N*)	Lower middle MPR (*N*)	Upper middle MPR (*N*)	High MPR (*N*)
Amitriptyline 25 mg cap/tab	Procurement	public	1.28 (7)	1.78 (24)	1.91 (13)	2.07 (4)
	Patient	public	3.06 (5)	3.18 (21)	4.16 (13)	1.81 (4)
		Private	12.28 (9)	16.60 (22)	15.60 (16)	24.45 (3)
Fluoxetine 20 mg cap/tab	Procurement	public	2.02 (3)	15.98 (11)	6.18 (7)	11.08 (6)
	Patient	public	3.59 (3)	35.16 (12)	28.01 (7)	14.49 (7)
		Private	26.32 (6)	65.21 (19)	72.54 (12)	100.24 (6)
Diazepam 5 mg cap/tab	Procurement	public	1.01 (8)	3.49 (21)	1.43 (7)	8.60 (5)
	Patient	public	9.69 (7)	4.10 (19)	2.99 (7)	0.00 (7)
		Private	16.64 (10)	20.82 (21)	25.81 (6)	23.98 (3)

*N*, number of countries; MPR, median price ratio.

Affordability, expressed as a median number of days of work to purchase a defined course of treatment, is presented in Table 3 for lowest-price generic psychotropic medicines, and in the online supplement for originator psychotropic medicines. For generics, affordability was higher in the public than the private sector, and in high-income countries than LMICs. Affordability analysis by WHO Region did not show any clear pattern (online supplement).

**Table 3.** Affordability of lowest-price generic psychotropic medicines in the public and private sector by income level, expressed as mean number of day's wages of the lowest-paid unskilled government worker needed to purchase a month of treatment

Psychotropic medicine	Sector	Income Level			
		Low days (*N*)	Lower middle days (*N*)	Upper middle days (*N*)	High days (*N*)
Amitriptyline 25 mg cap/tab	Public Private	1.3 (6) 2.2 (10)	1.3 (9) 1.7 (25)	0.4 (9) 0.8 (14)	0.2 (1) 1.1 (5)
Fluoxetine 20 mg cap/tab	Public Private	1.4 (2) 2.2 (4)	0.8 (2) 3.1 (10)	4.0 (5) 5.1 (9)	0.1 (1) 3.1 (5)
Carbamazepine 200 mg cap/tab	Public Private	NA 4.6 (1)	NA 0.5 (1)	0.2 (1) 0.6 (2)	NA NA
Diazepam 5 mg cap/tab	Public Private	0.1 (5) 0.2 (8)	0.1 (4) 0.2 (13)	0.0 (3) 0.1 (5)	0.0 (1) 0.0 (1)

*N*, number of countries; NA, not available.

## Discussion

The results of this study showed that the availability of essential psychotropic medicines in several countries, including many low- and middle-income countries, is poor. The average availability of generic and originator psychotropic medicines in the public sector was below 50% for all medicines, with low-income countries showing rates lower than the overall average in most cases. For most psychotropic medicines, availability was higher in the private sector, and for generic than originator formulations. Interestingly, in low-income countries availability of generic formulations in the private sector was higher than the average overall availability, reaching values of 50% or above for all medicines. In the private sector, however, psychotropic medicines were more expensive and less affordable, with around double the mean days of work to purchase a month of treatment as compared with the public sector. In low-income countries, psychotropic medicines were less affordable than in countries belonging to other income levels.

These findings are consistent with similar analyses carried out on other groups of medicines. Cameron and colleagues, for example, found that antiepileptic availability was higher in the private than the public sector, with low overall rates of availability and poor affordability, especially in low-income countries (Cameron *et al*., 2012). Similarly, Husain and colleagues, who conducted an analysis of 12 cardiovascular disease/hypertension medications, identified low availability and high costs as major barriers to the use of essential cardiovascular disease and antihypertensive medications worldwide, particularly in low- and middle-income countries (Husain *et al*., 2020). In terms of the medicine selection process, consistently with our findings, a recent scoping review on the use of the essential medicines concept found that countries varied greatly in the alignment of national EMLs to the WHO EML (Mahmic-Kaknjo *et al*., 2018). We additionally found that the proportion of countries that included in national EMLs medicines recently added to the WHO list, such as risperidone or clozapine, was extremely low, but, surprisingly, low proportions were also found for medicines that have been in the list for over 40 years, such as lithium or long-acting fluphenazine, especially in low-income countries. A clear pattern linking lack of selection, poor availability, high costs and poor affordability was identified, resulting in poor overall access to essential psychotropic medicines in many world countries.

The present analysis has some limitations that should be considered in interpreting the data. First, of the 192 WHO member states, the WHO repository included medicine selection data for 112 countries, while for availability, affordability and prices the number of countries with usable data varied for each psychotropic medicine. In some cases, we found only a small number of countries with data on certain psychotropic medicines. Therefore, the average estimates that we calculated cannot be generalised to all countries belonging to a geographical area or to an income level, as it is likely that the availability of survey data was not at random. It may be speculated that countries with availability or affordability problems were more likely to be the object of a survey, aiming to identify and characterise challenging issues in medicines selection, availability, or affordability. It is important to note, however, that these results may be used to identify a number of country-specific access problems, irrespective of geographical area or income level, and to highlight some overarching cross-country challenging issues in medicine access. Even some high-income countries showed huge gaps that limit access to essential psychotropic medicines to those in need. What can be generalised, therefore, is that available evidence suggests that no countries can claim to be fully aligned with the general principle of providing full access to essential psychotropic medicines.

A second group of limitations refers to the WHO/HAI methodology (HAI, 2021). Survey results reflected the availability of individual products on the day of data collection, and not average availability over time. Given that the treatment of mental health conditions depends upon the continuous supply of psychotropic medicines, it would have been useful to have data on the consistency in supply over time, particularly in the public sector. Additionally, availability results are also specific to the individual formulation and strength included in the survey. Therefore, the availability of other common strengths of the same medicine was not surveyed. However, medicines included in the surveys were selected to reflect medicines commonly used worldwide as well as those selected nationally based on disease and consumption patterns, and should therefore provide a reasonable estimate of overall availability.

A third limitation is that countries that did not select essential psychotropic medicines included in the WHO EML, or countries that do not have a national EML, should not be considered without any medicines for mental health conditions. For example, countries that have not included long-acting fluphenazine may have included another long-acting formulation, for example a second-generation long-acting antipsychotic, based on efficacy, tolerability and cost-effectiveness considerations. Of course, these countries did not follow the WHO EML, but still they followed a selection process and included a medicine that was considered essential. This consideration highlights that if many countries did not adhere to the WHO EML, it may possibly imply that the mental health chapter of the WHO EML is not fully aligned with current best evidence. We note that over the last 10 years the WHO has made a tremendous effort to produce, and regularly update, a number of evidence-based tools in the area of mental health, including recommendations (Barbui *et al*., 2010, 2015; Dua *et al*., 2011, 2016; Das-Munshi *et al*., 2020; Gronholm *et al*., 2021), evidence-based intervention guides (Keynejad *et al*., 2018), and related implementation and operational manuals. These tools include up-to-date recommendations on the selection and rational use of psychotropic medicines that not always fully match with the essential psychotropic medicines included in the WHO EML. Some essential psychotropic medicines may no longer be essential, as they were included in the list more than 40 years ago, when the first WHO EML was published. Aligning the WHO EML with existing WHO recommendations and tools, and with the current best evidence, would probably induce more countries to fully adhere to the WHO list.

The present findings, by showing that access to essential psychotropic medicines is limited in several countries at different economic development, call for urgent actions (Hogerzeil *et al*., 2013; Barbui *et al*., 2017). Countries should implement and maintain a reliable, accountable and transparent selection process, aiming to select a limited number of psychotropic medicines based on current best evidence, as well as to define which of these are essential, using the WHO EML as a reference standard. In parallel, the WHO EML should be updated to better reflect the current best pharmacological treatments for mental health conditions. Once a reliable selection process is functioning, an efficient system for procuring, storing and distributing medicines should be developed to ensure effective supplies. Technical instructions are available to support countries in conducting these crucial steps (WHO, 2021a, 2021b, 2021c, 2021d). As part of a supply system, a reliable quality control system should be implemented. As available psychotropic medicines should be affordable, sustainable financing systems should be developed. Competitive bulk procurement by generic name, for example, is considered a key policy in both developed and developing countries. The price to the end-users should also be monitored, as too often the taxes and duties levied on medicines, and the mark-ups made by dispensing doctors and pharmacies, result in huge differences between procurement and patient prices, as shown in this study.

Decision makers should consider that access to psychotropic medicines may be a proxy of access to mental healthcare. Mental Health Atlas 2020 reported that service coverage for mental health conditions is extremely low, with a global median of 29% of individuals with psychosis receiving mental health services (WHO, 2021e). Increasing service coverage at least by half is one of the core targets of the WHO Comprehensive Mental Health Action Plan 2013–2030 (WHO, 2021e), which recently recommended member states to ‘procure and ensure the availability of basic medicines for mental disorders included in the WHO Model List of Essential Medicines at all health system levels, ensure their rational use, and enable non-specialist health workers with adequate training to prescribe such medicines’ (WHO, 2021f). Therefore, improving access to psychotropic medicines may represent a unique chance for a transformative improvement of the whole mental healthcare system.

After 20 years from World Health Assembly resolution 54.11, which requested the WHO to coordinate the implementation of reliable systems to monitor access to essential medicines, with a view to improving access, finding reliable information on psychotropic medicine prices and availability remains a challenging issue. As collecting data and presenting it to governments can stimulate action, further research should be conducted to increase the number of countries with medicine availability, price and affordability data and to develop, implement and enforce policies that lower costs and increase availability and overall access.

## Data Availability

All data are available online as supplemental material of the present article.

## Supplementary material

For supplementary material accompanying this paper visit https://doi.org/10.1017/S2045796022000087. click here to view supplementary material
