# Supplementary material for: Mapping the selection, availability, price and affordability of essential medicines for mental health conditions at a global level
Source: Epidemiol Psychiatr Sci. 2022 Apr 19;31:e22. doi: 10.1017/S2045796022000087 (PMC9069582; doi:10.1017/S2045796022000087)
Supplement: Supplementary file 1 [file S2045796022000087sup001.docx]

Records screened
(n=918)

Records screened
(n=1204)

Records after duplicates removed
(n=1204)

Additional records identified through other sources
(n=3)

Records identified through database searching
(n=1432)

## Screening

## Identification

Full-text articles assessed for eligibility
(n=123)

Records excluded
(n=1081)

Full-text articles excluded:
(n=104)

WHO/HAI data not available (n=98); duplicates data stored in the WHO/HAI database (n=6)

## Eligibility

Studies included in review

(n=19)

(n=)

## Included

**PRISMA flow-diagram**

**References of included and excluded surveys**

| 1 | Adhikari, S. R., Pandey, A. R., Ghimire, M., Thapa, A. K. & Lamsal, D. K. (2018). Universal Access to Essential Medicines: An Evaluation of Nepal's Free Health Care Scheme. *J Nepal Health Res Counc* 16, 36-42. | WHO/HAI data not available |
| --- | --- | --- |
| 2 | Ahmad, N. S. & Islahudin, F. (2018). Affordability of essential medicine prices in Malaysia's private health sector. *Patient Prefer Adherence* 12, 1231-1237. | Included |
| 3 | Alefan, Q., Amairi, R. & Tawalbeh, S. (2018). Availability, prices and affordability of selected essential medicines in Jordan: a national survey. *BMC Health Serv Res* 18, 787. | Included |
| 4 | Alvares, J., Guerra, A. A. J., Araujo, V. E., Almeida, A. M., Dias, C. Z., Ascef, B. O., Costa, E. A., Guibu, I. A., Soeiro, O. M., Leite, S. N., Karnikowski, M. G. O., Costa, K. S. & Acurcio, F. A. (2017). Access to medicines by patients of the primary health care in the Brazilian Unified Health System. *Rev Saude Publica* 51, 20s. | WHO/HAI data not available |
| 5 | Armstrong-Hough, M., Kishore, S. P., Byakika, S., Mutungi, G., Nunez-Smith, M. & Schwartz, J. I. (2018). Disparities in availability of essential medicines to treat non-communicable diseases in Uganda: A Poisson analysis using the Service Availability and Readiness Assessment. *PLoS One* 13, e0192332. | WHO/HAI data not available |
| 6 | Armstrong-Hough, M., Sharma, S., Kishore, S. P., Akiteng, A. R. & Schwartz, J. I. (2020). Variation in the availability and cost of essential medicines for non-communicable diseases in Uganda: A descriptive time series analysis. *PLoS One* 15, e0241555. | WHO/HAI data not available |
| 7 | Ashigbie, P. G., Rockers, P. C., Laing, R. O., Cabral, H. J., Onyango, M. A., Buleti, J. P. L. & Wirtz, V. J. (2020). Availability and prices of medicines for non-communicable diseases at health facilities and retail drug outlets in Kenya: a cross-sectional survey in eight counties. *BMJ Open* 10, e035132. | WHO/HAI data not available |
| 8 | Atif, M., Malik, I., Mushtaq, I. & Asghar, S. (2019). Medicines shortages in Pakistan: a qualitative study to explore current situation, reasons and possible solutions to overcome the barriers. *BMJ Open* 9, e027028. | WHO/HAI data not available |
| 9 | Bandyopadhyay, S., Dutta, A. & Ghose, A. (2015). Access to Medicine in Public Hospitals and Some Crucial Management Issues. In *Developing Country Perspectives on Public Service Delivery* (ed. A. Gurtoo and C. Williams). Springer India: India. | WHO/HAI data not available |
| 10 | Barbi, L., Carvalho, L. & Luz, T. (2019). Antidepressivos, ansiolíticos, hipnóticos e sedativos: uma análise dos gastos em Minas Gerais / Antidepressive, antianxiety and hypnotics and sedatives agents: an analysis of public expenditure in Minas Gerais state, Brazil. *Physis (Rio J.)* 29, e290407. | WHO/HAI data not available |
| 11 | Bazargani, Y. T., Ewen, M., de Boer, A., Leufkens, H. G. & Mantel-Teeuwisse, A. K. (2014). Essential medicines are more available than other medicines around the globe. *PLoS One* 9, e87576. | WHO/HAI data not available |
| 12 | Beran, D., Pedersen, H. B. & Robertson, J. (2019). Noncommunicable diseases, access to essential medicines and universal health coverage. *Glob Health Action* 12, 1670014. | WHO/HAI data not available |
| 13 | Bermudez, J., Mendoza-Ruiz, A., Soares, S., Campos, M., Brum, A., Dantas, S. & Costa, J. (2018). Laudato Si': a bridge towards access to medicines. *Vigil. sanit. debate* 6, 3. | WHO/HAI data not available |
| 14 | Birabwa, C., Murison, J., Evans, V., Obua, C., Agaba, A., Waako, P. & Pollock, A. (2014). The availability of six tracer medicines in private medicine outlets in Uganda. *J Pharm Policy Pract* 7, 18. | Included |
| 15 | Bizimana, T., Kayumba, P. C. & Heide, L. (2020). Prices, availability and affordability of medicines in Rwanda. *PLoS One* 15, e0236411. | Included |
| 16 | Brhlikova, P., Maigetter, K., Murison, J., Agaba, A. G., Tusiimire, J. & Pollock, A. M. (2020). Registration and local production of essential medicines in Uganda. *J Pharm Policy Pract* 13, 31. | WHO/HAI data not available |
| 17 | Broccoli, M. C., Pigoga, J. L., Nyirenda, M., Wallis, L. A. & Calvello Hynes, E. J. (2018). Essential medicines for emergency care in Africa. *Afr J Emerg Med* 8, 110-117. | WHO/HAI data not available |
| 18 | Cardins, K., Freitas, C., Simões, M. & Costa, G. (2019). Access and rational use of medicines in the prison system in Paraíba. *Esc. Anna Nery Rev. Enferm* 23, e20180317. | WHO/HAI data not available |
| 19 | Chahal, H. S., St Fort, N. & Bero, L. (2013). Availability, prices and affordability of essential medicines in Haiti. *J Glob Health* 3, 020405. | Same data stored in the WHO/HAI repository |
| 20 | Chen, C., Yun, L., Dandan, A. & Tingting, W. (2019). Analysis of the Accessibility of Common Essential Medicine for 6 Kinds of Chronic Disease in Primary Health Care Institutions in Hubei Province from 2015 to 2017 / 中国药房. *China Pharmacy* 12, 5-10. | WHO/HAI data not available |
| 21 | Chen, W., Tang, S., Sun, J., Ross-Degnan, D. & Wagner, A. K. (2010). Availability and use of essential medicines in China: manufacturing, supply, and prescribing in Shandong and Gansu provinces. *BMC Health Serv Res* 10, 211. | WHO/HAI data not available |
| 22 | Dabare, P. R., Wanigatunge, C. A. & Beneragama, B. H. (2014). A national survey on availability, price and affordability of selected essential medicines for non communicable diseases in Sri Lanka. *BMC Public Health* 14, 817. | More complete/recent data stored in the WHO/HAI repository |
| 23 | Dal Pizzol Tda, S., Trevisol, D. J., Heineck, I., Flores, L. M., Camargo, A. L., Koenig, A., Torres, I. L., Kadri, M. C., Monreal, M. T., Melo, A. M. & Ferreira, M. B. (2010). [Adherence to essential medicines in cities from three Brazilian states]. *Cad Saude Publica* 26, 827-36. | WHO/HAI data not available |
| 24 | Dixit, R., Vinay, M., Jayasree, T., Ubedulla, S., Manohar, V. S. & Chandrasekhar, N. (2011). Availability of essential medicines: A primary health care perspective. *Indian J Pharmacol* 43, 599-600. | WHO/HAI data not available |
| 25 | Dong, Z., Tao, Q., Yan, B. & Sun, G. (2020). Availability, prices and affordability of essential medicines in Zhejiang Province, China. *PLoS One* 15, e0241761. | Included |
| 26 | Dorj, G., Sunderland, B., Sanjjav, T., Dorj, G. & Gendenragchaa, B. (2017). Drug pricing and reimbursement decision making systems in Mongolia. *J Pharm Policy Pract* 10, 11. | WHO/HAI data not available |
| 27 | Droti, B., O'Neill, K. P., Mathai, M., Yao Tsidi Dovlo, D. & Robertson, J. (2019). Poor availability of essential medicines for women and children threatens progress towards Sustainable Development Goal 3 in Africa. *BMJ Glob Health* 4, e001306. | WHO/HAI data not available |
| 28 | Dutra, K., Martins, U. & Lima, M. (2016). Availability and Accessibility to Medicines in Primary Health Care in a Brazilian Region. *J Young Pharm* 8, 255-258. | WHO/HAI data not available |
| 29 | Emmanuel Awucha, N., Chinelo Janefrances, O., Chima Meshach, A., Chiamaka Henrietta, J., Ibilolia Daniel, A. & Esther Chidiebere, N. (2020). Impact of the COVID-19 Pandemic on Consumers' Access to Essential Medicines in Nigeria. *Am J Trop Med Hyg* 103, 1630-1634. | WHO/HAI data not available |
| 30 | Ewen, M., Zweekhorst, M., Regeer, B. & Laing, R. (2017). Baseline assessment of WHO's target for both availability and affordability of essential medicines to treat non-communicable diseases. *PLoS One* 12, e0171284. | WHO/HAI data not available |
| 31 | Fang, Y., Wagner, A. K., Yang, S., Jiang, M., Zhang, F. & Ross-Degnan, D. (2013). Access to affordable medicines after health reform: evidence from two cross-sectional surveys in Shaanxi Province, western China. *Lancet Glob Health* 1, e227-37. | Same data stored in the WHO/HAI repository |
| 32 | Ferrario, A., Chitan, E., Seicas, R., Sautenkova, N., Bezverhni, Z., Kluge, H. & Habicht, J. (2016). Progress in increasing affordability of medicines for non-communicable diseases since the introduction of mandatory health insurance in the Republic of Moldova. *Health Policy Plan* 31, 793-800. | WHO/HAI data not available |
| 33 | Ferreira-Filho, J., GT, C. & Mastroianni, P. (2010). Acesso a medicamentos essenciais em farmácias e drogarias do Município de Araraquara. *Revista de Ciências Farmacêuticas Básica e Aplicada* 31, 177-182. | WHO/HAI data not available |
| 34 | Figueiredo, T. A., Schramm, J. M. & Pepe, V. L. (2014). [Selection of essential medicines and the burden of disease in Brazil.]. *Cad Saude Publica* 30, 2344-2356. | WHO/HAI data not available |
| 35 | Fulone, I., Barberato-Filho, S., dos Santos, M. F., Rossi Cde, L., Guyatt, G. & Lopes, L. C. (2016). Essential psychiatric medicines: wrong selection, high consumption and social problems. *BMC Public Health* 16, 52. | WHO/HAI data not available |
| 36 | Guan, X., Hu, H., Man, C. & Shi, L. (2018). A survey of availability, price and affordability of essential medicines from 2011 to 2016 in Chinese secondary and tertiary hospitals. *Int J Equity Health* 17, 158. | WHO/HAI data not available |
| 37 | Gupta, N., Coates, M. M., Bekele, A., Dupuy, R., Fenelon, D. L., Gage, A. D., Getachew, T., Karmacharya, B. M., Kwan, G. F., Lulebo, A. M., Masiye, J. K., Mayige, M. T., Ndour Mbaye, M., Mridha, M. K., Park, P. H., Dagnaw, W. W., Wroe, E. B. & Bukhman, G. (2020). Availability of equipment and medications for non-communicable diseases and injuries at public first-referral level hospitals: a cross-sectional analysis of service provision assessments in eight low-income countries. *BMJ Open* 10, e038842. | WHO/HAI data not available |
| 38 | Gupta, R., Bollyky, T. J., Cohen, M., Ross, J. S. & Kesselheim, A. S. (2018). Affordability and availability of off-patent drugs in the United States-the case for importing from abroad: observational study. *BMJ* 360, k831. | WHO/HAI data not available |
| 39 | Hailu, A. D. & Mohammed, S. A. (2020). Availability, price, and affordability of WHO priority maternal and child health medicine in public health facilities of Dessie, north-East Ethiopia. *BMC Med Inform Decis Mak* 20, 221. | WHO/HAI data not available |
| 40 | Haque, M. (2017). Essential Medicine Utilization and Situation in Selected Ten Developing Countries: A Compendious Audit. *J Int Soc Prev Community Dent* 7, 147-160. | WHO/HAI data not available |
| 41 | Hong, L. & Ye, H. (2017). Establishment of Evaluation Index System for the Implementation of National Essential Medicine System in Community Health Service Centers in Shanghai / 中国药房. *China Pharmacy* 12, 3321-3325. | WHO/HAI data not available |
| 42 | Huang, Y., Jiang, Y., Zhang, L., Mao, W., van Boven, J. F. M., Postma, M. J. & Chen, W. (2018). Availability, use, and affordability of medicines in urban China under universal health coverage: an empirical study in Hangzhou and Baoji. *BMC Health Serv Res* 18, 218. | WHO/HAI data not available |
| 43 | Huang, Z., Cao, Y. & Li, H. (2017). Construction of Essential Medicine Selection Model Based on Knapsack Theory / 中国药房. *China Pharmacy* 12, 2017-2020. | WHO/HAI data not available |
| 44 | Hunie, M., Desse, T., Fenta, E., Teshome, D., Gelaw, M. & Gashaw, A. (2020). Availability of Emergency Drugs and Essential Equipment in Intensive Care Units in Hospitals of Ethiopia: A Multicenter Cross-Sectional Study. *Open Access Emerg Med* 12, 435-440. | WHO/HAI data not available |
| 45 | Jangra, S. & Gilhotra, N. (2015). Comparative Availability of Selected Essential Medicines for Selected Chronic Diseases in Bhiwani District, Haryana, India. *Journal of Applied Pharmaceutical Science* 5, 131-138. | Included |
| 46 | Jarvis, J. D., Woods, H., Bali, A., Oronsaye, E. & Persaud, N. (2019). Selection of WHO-recommended essential medicines for non-communicable diseases on National Essential Medicines Lists. *PLoS One* 14, e0220781. | WHO/HAI data not available |
| 47 | Jiang, M., Zhou, Z., Wu, L., Shen, Q., Lv, B., Wang, X., Yang, S. & Fang, Y. (2015). Medicine prices, availability, and affordability in the Shaanxi Province in China: implications for the future. *Int J Clin Pharm* 37, 12-7. | Same data stored in the WHO/HAI repository |
| 48 | Jiang, X., Wang, Y., Jia, S., Gao, Y. & Sun, L. (2019). Evaluation of Essential Medicines Accessibility in Liaoning Province. *Chinese Pharmaceutical Journal* 6, 501-505. | WHO/HAI data not available |
| 49 | Joshua, I. B., Passmore, P. R. & Sunderland, B. V. (2016). An evaluation of the Essential Medicines List, Standard Treatment Guidelines and prescribing restrictions, as an integrated strategy to enhance quality, efficacy and safety of and improve access to essential medicines in Papua New Guinea. *Health Policy Plan* 31, 538-46. | WHO/HAI data not available |
| 50 | K, C. B., Heydon, S. & Norris, P. (2015). Access to and quality use of non-communicable diseases medicines in Nepal. *J Pharm Policy Pract* 8, 21. | WHO/HAI data not available |
| 51 | Kasonde, L., Tordrup, D., Naheed, A., Zeng, W., Ahmed, S. & Babar, Z. U. (2019). Evaluating medicine prices, availability and affordability in Bangladesh using World Health Organisation and Health Action International methodology. *BMC Health Serv Res* 19, 383. | Included |
| 52 | Katabalo, D., Hamasaki, K., Melkisedeck, J. & Mwita, S. (2019). Availability of Selected Essential Prescription Medicines and Trained Dispensers in Accredited Drug Dispensing Outlets in Tanzania. A Case Study of Hanang District Council in Manyara Region Northern Tanzania. *International Journal of Contemporary Medical Research* 6, 1-5. | WHO/HAI data not available |
| 53 | Kefale, A. T. & Shebo, H. H. (2019). Availability of essential medicines and pharmaceutical inventory management practice at health centers of Adama town, Ethiopia. *BMC Health Serv Res* 19, 254. | WHO/HAI data not available |
| 54 | Khanal, S., Veerman, L., Ewen, M., Nissen, L. & Hollingworth, S. (2019). Availability, Price, and Affordability of Essential Medicines to Manage Noncommunicable Diseases: A National Survey From Nepal. *Inquiry* 56, 46958019887572. | Included |
| 55 | Khuluza, F. & Haefele-Abah, C. (2019). The availability, prices and affordability of essential medicines in Malawi: A cross-sectional study. *PLoS One* 14, e0212125. | Included |
| 56 | Kibira, D., Kitutu, F. E., Merrett, G. B. & Mantel-Teeuwisse, A. K. (2017). Availability, prices and affordability of UN Commission's lifesaving medicines for reproductive and maternal health in Uganda. *J Pharm Policy Pract* 10, 35. | WHO/HAI data not available |
| 57 | Kotwani, A. (2012). Psychiatric medicines in India: why public healthcare facilities and a thriving generics industry cannot assure access and affordability. *Int Psychiatry* 9, 34-36. | WHO/HAI data not available |
| 58 | Kotwani, A. (2013). Where are we now: assessing the price, availability and affordability of essential medicines in Delhi as India plans free medicine for all. *BMC Health Serv Res* 13, 285. | Same data stored in the WHO/HAI repository |
| 59 | Kristina, S., Aditama, H., Endarti, D. & Widayanti, A. (2020). Evaluating Accessibility of Essential Medicines in Indonesia: A Survey on Availability and Prices in Public and Private Health Sectors. *International Journal of Pharmaceutical Research* 12, 692-699. | Included |
| 60 | Kuwawenaruwa, A., Wyss, K., Wiedenmayer, K., Metta, E. & Tediosi, F. (2020). The effects of medicines availability and stock-outs on household's utilization of healthcare services in Dodoma region, Tanzania. *Health Policy Plan* 35, 323-333. | WHO/HAI data not available |
| 61 | Lambojon, K., Chang, J., Saeed, A., Hayat, K., Li, P., Jiang, M., Atif, N., Desalegn, G. K., Khan, F. U. & Fang, Y. (2020). Prices, Availability and Affordability of Medicines with Value-Added Tax Exemption: A Cross-Sectional Survey in the Philippines. *Int J Environ Res Public Health* 17. | WHO/HAI data not available |
| 62 | Mao, W., Huang, Y. & Chen, W. (2019). An analysis on rational use and affordability of medicine after the implementation of National Essential Medicines Policy and Zero Mark-up Policy in Hangzhou, China. *PLoS One* 14, e0213638. | WHO/HAI data not available |
| 63 | Masters, S. H., Burstein, R., DeCenso, B., Moore, K., Haakenstad, A., Ikilezi, G., Achan, J., Osei, I., Garshong, B., Kisia, C., Njuguna, P., Babigumira, J., Kumar, S., Hanlon, M. & Gakidou, E. (2014). Pharmaceutical availability across levels of care: evidence from facility surveys in Ghana, Kenya, and Uganda. *PLoS One* 9, e114762. | WHO/HAI data not available |
| 64 | Mendis, S., Fukino, K., Cameron, A., Laing, R., Filipe, A., Jr., Khatib, O., Leowski, J. & Ewen, M. (2007). The availability and affordability of selected essential medicines for chronic diseases in six low- and middle-income countries. *Bull World Health Organ* 85, 279-88. | WHO/HAI data not available |
| 65 | Mhlanga, B. S. & Suleman, F. (2014). Price, availability and affordability of medicines. *Afr J Prim Health Care Fam Med* 6, E1-6. | Included |
| 66 | Millar, T. P., Wong, S., Odierna, D. H. & Bero, L. A. (2011). Applying the essential medicines concept to US preferred drug lists. *Am J Public Health* 101, 1444-8. | WHO/HAI data not available |
| 67 | Millard, C., Kadam, A. B., Mahajan, R., Pollock, A. M. & Brhlikova, P. (2018). Availability of brands of six essential medicines in 124 pharmacies in Maharashtra. *J Glob Health* 8, 010402. | Included |
| 68 | Minaei, H., Peikanpour, M., Yousefi, N., Peymani, P., Peiravian, F., Shobeiri, N., Karimi Majd, Z. & Shamsaee, J. (2019). Country Pharmaceutical Situation on Access, Quality, and Rational Use of Medicines: An Evidence from a middle-income country. *Iran J Pharm Res* 18, 2191-2203. | WHO/HAI data not available |
| 69 | Modisakeng, C., Matlala, M., Godman, B. & Meyer, J. C. (2020). Medicine shortages and challenges with the procurement process among public sector hospitals in South Africa; findings and implications. *BMC Health Serv Res* 20, 234. | WHO/HAI data not available |
| 70 | Mohamed Ibrahim, M. I., Alshakka, M., Al-Abd, N., Bahattab, A. & Badulla, W. (2020). Availability of Essential Medicines in a Country in Conflict: A Quantitative Insight from Yemen. *Int J Environ Res Public Health* 18. | WHO/HAI data not available |
| 71 | Mori, A. T., Kaale, E. A., Ngalesoni, F., Norheim, O. F. & Robberstad, B. (2014). The role of evidence in the decision-making process of selecting essential medicines in developing countries: the case of Tanzania. *PLoS One* 9, e84824. | WHO/HAI data not available |
| 72 | Moye-Holz, D., van Dijk, J. P., Reijneveld, S. A. & Hogerzeil, H. V. (2017). Policy approaches to improve availability and affordability of medicines in Mexico - an example of a middle income country. *Global Health* 13, 53. | WHO/HAI data not available |
| 73 | Mukundiyukuri, J. P., Irakiza, J. J., Nyirahabimana, N., Ng'ang'a, L., Park, P. H., Ngoga, G., El-Khatib, Z., Nditunze, L., Dusengeyezu, E., Rusangwa, C., Mpunga, T., Mubiligi, J. & Hedt-Gauthier, B. (2020). Availability, Costs and Stock-Outs of Essential NCD Drugs in Three Rural Rwandan Districts. *Ann Glob Health* 86, 123. | WHO/HAI data not available |
| 74 | Mustafa, A. A. & Kowalski, S. R. (2010). A comparative analysis of the Libyan national essential medicines list and the WHO model list of essential medicines. *Libyan J Med* 5. | WHO/HAI data not available |
| 75 | Nascimento, R., Alvares, J., Guerra, A. A. J., Gomes, I. C., Costa, E. A., Leite, S. N., Costa, K. S., Soeiro, O. M., Guibu, I. A., Karnikowski, M. G. O. & Acurcio, F. A. (2017). Availability of essential medicines in primary health care of the Brazilian Unified Health System. *Rev Saude Publica* 51, 10s. | WHO/HAI data not available |
| 76 | Oliveira, M. A., Luiza, V. L., Tavares, N. U., Mengue, S. S., Arrais, P. S., Farias, M. R., Pizzol, T. D., Ramos, L. R. & Bertoldi, A. D. (2016). Access to medicines for chronic diseases in Brazil: a multidimensional approach. *Rev Saude Publica* 50, 6s. | WHO/HAI data not available |
| 77 | Ozawa, S., Shankar, R., Leopold, C. & Orubu, S. (2019). Access to medicines through health systems in low- and middle-income countries. *Health Policy Plan* 34, iii1-iii3. | WHO/HAI data not available |
| 78 | Paunikar, A. & Bhave, K. (2015). Cost analysis of oral antidepressant drugs available in India. *National Journal of Physiology, Pharmacy and Pharmacology* 5, 367-371. | WHO/HAI data not available |
| 79 | Pekez-Pavlisko, T., Racic, M. & Kusmuk, S. (2017). Medicine Availability and Prescribing Policy for Non-Communicable Diseases in the Western Balkan Countries. *Front Public Health* 5, 295. | WHO/HAI data not available |
| 80 | Perehudoff, S. K., Alexandrov, N. V. & Hogerzeil, H. V. (2019a). Access to essential medicines in 195 countries: A human rights approach to sustainable development. *Glob Public Health* 14, 431-444. | WHO/HAI data not available |
| 81 | Perehudoff, S. K., Alexandrov, N. V. & Hogerzeil, H. V. (2019b). The right to health as the basis for universal health coverage: A cross-national analysis of national medicines policies of 71 countries. *PLoS One* 14, e0215577. | WHO/HAI data not available |
| 82 | Perumal-Pillay, V. A. & Suleman, F. (2017). Selection of essential medicines for South Africa - an analysis of in-depth interviews with national essential medicines list committee members. *BMC Health Serv Res* 17, 17. | WHO/HAI data not available |
| 83 | Perumal-Pillay, V. A. & Suleman, F. (2020). Understanding the decision making process of selection of medicines in the private sector in South Africa - lessons for low-middle income countries. *J Pharm Policy Pract* 13, 17. | WHO/HAI data not available |
| 84 | Pinto Cdu, B., Miranda, E. S., Emmerick, I. C., Costa Ndo, R. & Castro, C. G. (2010). Medicine prices and availability in the Brazilian Popular Pharmacy Program. *Rev Saude Publica* 44, 611-9. | WHO/HAI data not available |
| 85 | Prinja, S., Bahuguna, P., Tripathy, J. P. & Kumar, R. (2015). Availability of medicines in public sector health facilities of two North Indian States. *BMC Pharmacol Toxicol* 16, 43. | Included |
| 86 | Rathish, D., Premarathna, I., Jayathilake, T., Kandegedara, C., Punchihewa, K., Ananda, L., Bandara, T., Jayasumana, C. & Siribaddana, S. (2017). Availability of essential medicines in selected public, primary and secondary health care institutions of a rural Sri Lankan district: a spot survey. *BMC Health Serv Res* 17, 11. | WHO/HAI data not available |
| 87 | Robertson, J., Mace, C., Forte, G., de Joncheere, K. & Beran, D. (2015). Medicines availability for non-communicable diseases: the case for standardized monitoring. *Global Health* 11, 18. | WHO/HAI data not available |
| 88 | Rockers, P. C., Laing, R. O., Ashigbie, P. G., Onyango, M. A., Mukiira, C. K. & Wirtz, V. J. (2019). Effect of Novartis Access on availability and price of non-communicable disease medicines in Kenya: a cluster-randomised controlled trial. *Lancet Glob Health* 7, e492-e502. | WHO/HAI data not available |
| 89 | Saeed, A., Saeed, H., Saleem, Z., Fang, Y. & Babar, Z. U. (2019). Evaluation of prices, availability and affordability of essential medicines in Lahore Division, Pakistan: A cross-sectional survey using WHO/HAI methodology. *PLoS One* 14, e0216122. | Included |
| 90 | Saeed, A., Saeed, H., Saleem, Z., Yang, C., Jiang, M., Zhao, M., Ji, W., Aziz, M. M., Khan, F. U., Gillani, A. H., Atif, N., Fang, Y. & Babar, Z. U. D. (2020). Impact of National Drug Pricing Policy 2018 on access to medicines in Lahore division, Pakistan: a pre-post survey study using WHO/HAI methodology. *BMJ Open* 10, e034720. | WHO/HAI data not available |
| 91 | Saiyoki, L., Rose, O. & Aggrey, O. (2019). Accessibility of essential medicines for non-communicable diseases in a devolved system of government in trans nzoia County, Kenya. *International Journal of Development Research* 5, 31586-315900. | WHO/HAI data not available |
| 92 | Salvi, M., Mezadri, T., Lacerda, L. & Grillo, L. (2018). Analysis of essential drugs lists of a regional healthcare coordination center in Rio Grande do Sul. *Mundo saúde (Impr.)* 42, 39-60. | WHO/HAI data not available |
| 93 | Saouadogo, H. & Compaore, M. (2010). Essential medicines access survey in public hospitals of Burkina Faso. *African Journal of Pharmacy and Pharmacology* 4, 373-380. | WHO/HAI data not available |
| 94 | Sarangi, S. C., Kaur, N., Tripathi, M. & Gupta, Y. K. (2018). Cost analysis study of neuropsychiatric drugs: Role of National List of Essential Medicines, India. *Neurol India* 66, 1427-1433. | WHO/HAI data not available |
| 95 | Senarathna, S. M., Mannapperuma, U. & Fernandopulle, B. M. (2011). Medicine prices, availability and affordability in Sri Lanka. *Indian J Pharmacol* 43, 60-3. | Included |
| 96 | Sengxeu, N., Dufat, H., Boumediene, F., Vorachit, S., Chivorakoun, P., Souvong, V., Manithip, C., Preux, P. M., Ratsimbazafy, V. & Jost, J. (2020). Availability, affordability, and quality of essential antiepileptic drugs in Lao PDR. *Epilepsia Open* 5, 550-561. | WHO/HAI data not available |
| 97 | Sharma, A., Rorden, L., Ewen, M. & Laing, R. (2016). Evaluating availability and price of essential medicines in Boston area (Massachusetts, USA) using WHO/HAI methodology. *J Pharm Policy Pract* 9, 12. | More complete/recent data stored in the WHO/HAI repository |
| 98 | Shrestha, R., Ghale, A., Chapagain, B. R., Gyawali, M. & Acharya, T. (2017). Survey on the availability, price and affordability of selected essential medicines for non-communicable diseases in community pharmacies of Kathmandu valley. *SAGE Open Med* 5, 2050312117738691. | WHO/HAI data not available |
| 99 | Shukla, A. & Sharma, P. (2016). Cost variation study of antidepressant drugs. *International Journal of Basic & Clinical Pharmacology* 5, 1816-1821. | WHO/HAI data not available |
| 100 | Sieleunou, I., Turcotte-Tremblay, A. M., De Allegri, M., Taptue Fotso, J. C., Azinyui Yumo, H., Magne Tamga, D. & Ridde, V. (2019). How does performance-based financing affect the availability of essential medicines in Cameroon? A qualitative study. *Health Policy Plan* 34, iii4-iii19. | WHO/HAI data not available |
| 101 | Song, Y., Bian, Y. & Zhen, T. (2018). Making medicines more accessible in China: An empirical study investigating the early progress of essential medicine system. *PLoS One* 13, e0201582. | WHO/HAI data not available |
| 102 | Starling, F., Camargos, E., Ferreira, F., Freitas, M., Costa, J. & Medeiros-Souza, P. (2019). Unavailability of appropriate doses and need for tablet splitting of psychotropic drugs by geriatric patients. *Geriatr., Gerontol. Aging (Impr.)* 13, 126-132. | WHO/HAI data not available |
| 103 | Tang, Y., Liu, C. & Zhang, X. (2017). Delivery of Essential Medicines to Primary Care Institutions and its Association with Procurement Volume and Price: A Case Study in Hubei Province, China. *Appl Health Econ Health Policy* 15, 57-64. | WHO/HAI data not available |
| 104 | Thome, J., Marenah, E., Moraru, D., Hoppner, J. & Nyan, O. (2011). Availability of psychiatric medication in an urban area of The Gambia/West Africa. *World J Biol Psychiatry* 12 Suppl 1, 114-7. | Included |
| 105 | Thuy, H. T., Ha, D. T., Tuong, P. V., Thinh, C. Q. & Nga, N. T. (2020). Availability of essential medicines in primary care in Vietnam. *International Journal of Healthcare Management*. | WHO/HAI data not available |
| 106 | Tian, B., Hu, J., Hu, R. & Li, Z. (2017). Application of Analytic Hierarchy Process in Evaluation Index System of Additional Essential Medicine / 中国药房. *China Pharmacy* 12, 2881-2884. | WHO/HAI data not available |
| 107 | Tripathi, N., Kerketta, F., Chatterjee, P., Raman, V. R., John, D. & Jain, K. (2018). Access and availability of essential medicines in Chhattisgarh: Situation in public health facilities. *J Family Med Prim Care* 7, 152-156. | WHO/HAI data not available |
| 108 | Vialle-Valentin, C. E., Serumaga, B., Wagner, A. K. & Ross-Degnan, D. (2015). Evidence on access to medicines for chronic diseases from household surveys in five low- and middle-income countries. *Health Policy Plan* 30, 1044-52. | WHO/HAI data not available |
| 109 | Vogler, S., Schneider, P., Dedet, G. & Bak Pedersen, H. (2019). Affordable and equitable access to subsidised outpatient medicines? Analysis of co-payments under the Additional Drug Package in Kyrgyzstan. *Int J Equity Health* 18, 89. | WHO/HAI data not available |
| 110 | Wagenaar, B. H., Stergachis, A., Rao, D., Hoek, R., Cumbe, V., Napua, M. & Sherr, K. (2015). The availability of essential medicines for mental healthcare in Sofala, Mozambique. *Glob Health Action* 8, 27942. | WHO/HAI data not available |
| 111 | Wang, H., Sun, Q., Vitry, A. & Nguyen, T. A. (2017). Availability, Price, and Affordability of Selected Essential Medicines for Chronic Diseases in 11 Countries of the Asia Pacific Region: A Secondary Analysis. *Asia Pac J Public Health* 29, 268-277. | WHO/HAI data not available |
| 112 | Wang, W., Xiao, L., Li, C., Fang, X., Zhang, Y., Cui, D., Yin, X. & Mao, Z. (2018). Investigation on the Utilization of Essential Medicines in 26 Rural Primary Medical Institutions from Poverty-stricken Areas of Huanggang City / 中国药房. *China Pharmacy* 12, 156-159. | WHO/HAI data not available |
| 113 | Woldeyohanins, A., Meseret, B., Teka, M. & Teshome, T. (2020). Assessment of the availability of essential medicines and inventory control practice at university of Gondar comprehensive specialized hospital, Amhara regional state of Ethiopia: institutional based cross-sectional study design. *International Journal of Scientific Reports* 6, 349-352. | WHO/HAI data not available |
| 114 | Xi, X., Chen, P., Yang, F., Yang, Y., Chen, L. & Mao, N. (2018). Evaluating the accessibility of essential medicines in China. *J Med Econ* 21, 784-792. | WHO/HAI data not available |
| 115 | Xi, X., Li, W., Li, J., Zhu, X., Fu, C., Wei, X. & Chu, S. (2015). A survey of the availability, prices and affordability of essential medicines in Jiangsu Province, China. *BMC Health Serv Res* 15, 345. | Included |
| 116 | Xu, R., Li, S., Lv, X. & Xie, X. (2020). Prices, availability, and affordability of national essential medicines in public primary hospitals: A cross-sectional survey in poverty-stricken rural areas in China. *Int J Health Plann Manage* 35, 545-557. | Included |
| 117 | Yang, C., Hu, S., Ye, D., Jiang, M., Babar, Z. U. & Fang, Y. (2020). Evaluating Price and Availability of Essential Medicines in China: A Mixed Cross-Sectional and Longitudinal Study. *Front Pharmacol* 11, 602421. | Included |
| 118 | Yang, H., Dib, H. H., Zhu, M., Qi, G. & Zhang, X. (2010). Prices, availability and affordability of essential medicines in rural areas of Hubei Province, China. *Health Policy Plan* 25, 219-29. | WHO/HAI data not available |
| 119 | Yang, H., Xiao, J. & Hu, M. (2017). Construction of evaluation index system for evidence based selection of essential medicines in China / 中国卫生政策研究. *Chinese Journal of Health Policy* 12, 33-38. | WHO/HAI data not available |
| 120 | Zhang, H., Gu, S., Yang, Q., Gao, L., Zhen, X., Zeng, Y. & Dog, H. (2017). Evidence-based research on the selection method system for China's essential medicine list / 中华医院管理杂志. *Chinese Journal of Hospital Administration* 12, 293-297. | WHO/HAI data not available |
| 121 | Zhang, X., Wu, Q., Liu, G., Li, Y., Gao, L., Guo, B., Fu, W., Hao, Y., Cui, Y., Huang, W. & Coyte, P. C. (2014). The effect of the National Essential Medicines Policy on health expenditures and service delivery in Chinese township health centres: evidence from a longitudinal study. *BMJ Open* 4, e006471. | WHO/HAI data not available |
| 122 | Ziganshina, L., Razzakova, C., Hasan, S. & Babar, Z. (2020). Access to Medicines: Case Studies fromRussia and Kyrgyzstan. In *Global Pharmaceutical Policy*, pp. 223-246. Springer Singapore. | WHO/HAI data not available |
| 123 | Zuo, W., Sun, W., Tang, X., Niu, Z. & Zhang, B. (2020). Comparative Study on the Collection of Nervous System Medicines in Essential Medicine List of WHO and China / 中国药房. *China Pharmacy* 12, 397-401. | WHO/HAI data not available |

**Availability of essential psychotropic medicines by WHO Region and country income level**

|  | **Amitriptyline 25 mg cap tab** | **Fluoxetine 20 mg cap tab** | **Diazepam 5mg cap tab** | **Carbamazepine 200 mg cap tab** | **LAI fluphenazine 25 mg/ml inj** | **Risperidone 2 mg cap/tab** |
| --- | --- | --- | --- | --- | --- | --- |
|  | % (min; max) (n) | % (min; max) (n) | % (min; max) (n) | % (min; max) (n) | % (min; max) (n) | % (min; max) (n) |
|  |  |  |  |  |  |  |
| **Originator brand: public sector availability** | | | | | | |
| **All surveys** | 7.6 (0.0; 93.3) (57) | 10.4 (0.0; 85.0) (51) | 7.6 (0.0; 60.0) (55) | 17.7 (0.0; 100) (60) | 16.2 (0.0; 80.0) (26) | 1.7 (0.0; 10.0) (6) |
|  |  |  |  |  |  |  |
| **WHO REGION** |  |  |  |  |  |  |
| AFRO | 0.0 (0.0; 0.0) (10) | 0.0 (0.0; 0.0) (8) | 2.4 (0.0; 31.0) (13) | 3.4 (0.0; 9.5) (12) | 0.0 (0.0; 0.0) (6) | NA |
| EMRO | 27.2 (0.0; 93.3) (13) | 6.5 (0.0; 23.8) (12) | 23.4 (0.0; 60.0) (10) | 30.9 (0.0; 100) (14) | 66.8 (0.0; 80.0) (7) | 0.0 (0.0; 0.0) (3) |
| EURO | 0.0 (0.0; 0.0) (3) | 0.0 (0.0; 0.0) (1) | 0.0 (0.0; 0.0) (3) | 10.1 (0.0; 23.3) (4) | 0.0 (0.0; 0.0) (1) | NA |
| PAHO | 1.2 (0.0; 5.7) (11) | 0.3 (0.0; 0.3) (11) | 0.0 (0.0; 0.0) (8) | 44.9 (0.0; 40.4) (10) | 1.3 (0.0; 3.8) (3) | 3.3 (0.0; 10.0) (3) |
| SEARO | 0.3 (0.0; 5.0) (9) | 7.5 (0.0; 20.0) (9) | 0.1 (0.0; 1.2) (9) | 10.3 (0.0; 25.0) (8) | 0.0 (0.0; 0.0) (6) | NA |
| WPRO | 0.2 (0.0; 1.6) (9) | 18.6 (1.6; 85) (8) | 1.5 (0.0; 15.0) (10) | 33.6 (0.0; 95.0) (10) | 0.0 (0.0; 0.0) (2) | NA |
|  |  |  |  |  |  |  |
| **Income Level** |  |  |  |  |  |  |
| LIC | 13.0 (0.0; 90.9) (7) | 0.0 (0.0; 0.0) (4) | 0.0 (0.0; 0.0) (10) | 0.6 (0.0; 5.7) (9) | 0.0 (0.0; 0.0) (3) | 0.0 (0.0; 0.0) (1) |
| LMIC | 1.1 (0.0; 5.7) (23) | 4.4 (0.0; 23.8) (23) | 11.6 (0.0; 60.0) (24) | 12.6 (0.0; 50.0) (23) | 26.9 (0; 80) (15) | NA |
| UMIC | 1.2 (0.0; 5.6) (19) | 17.9 (0.0; 85) (18) | 5.9 (0.0; 33.0) (15) | 19.4 (0.0; 95.0) (21) | 1.7 (0.0; 3.8) (7) | 5 (0.0; 10.0) (2) |
| HIC | 21.5 (0.0; 93.3) (8) | 2.3 (0.0; 8.0) (6) | 7.0 (0.0; 22.2) (6) | 48.6 (0.0; 100) (8) | 0.0 (0.0; 0.0) (1) | 0 (0.0; 0.0) (3) |
|  |  |  |  |  |  |  |
| **Originator brand: private sector availability** | | | | | | |
| **All surveys** | 28.3 (0.0; 100) (65) | 27.1 (0.0; 100) (60) | 29.2 (0.0; 100) (62) | 52.3 (0.0; 100) (62) | 21.5 (0.0; 100) (25) | 47.4 (0.0; 90.0) (6) |
|  |  |  |  |  |  |  |
| **WHO REGION** |  |  |  |  |  |  |
| AFRO | 13.9 (0.0; 85.0) (14) | 20.5 (0.0; 46.7) (11) | 22.9 (0.0; 96.7) (14) | 54.1 (0.0; 90.0) (13) | 3.5 (0.0; 6.7) (6) | NA |
| EMRO | 52.6 (0.0; 100) (13) | 48.6 (0.0; 100) (14) | 47.4 (0.0; 100) (12) | 62.6 (0.0; 100) (15) | 47.9 (0.0; 100) (7) | 73.3 (60.0; 90.0) (3) |
| EURO | 0.0 (0.0; 0.0) (5) | 0.0 (0.0; 0.0) (3) | 0.0 (0.0; 0.0) (4) | 7.8 (0.0; 30.0) (5) | 0.0 (0.0; 0.0) (1) | NA |
| PAHO | 23.6 (0.0; 60.7) (11) | 14.9 (0.0; 33.3) (11) | 12.9 (0.0; 70.0) (10) | 51.8 (3.3; 96.7) (10) | 5.8 (0.0; 15.6) (3) | 21.4 (0.0; 52.4) (3) |
| SEARO | 58.1 (0.0; 90.0) (10) | 8.1 (0.0; 67.0) (10) | 46.9 (10.8; 92.5) (9) | 79.6 (34.4; 95.0) (8) | 0.0 (0.0; 0.0) (6) | NA |
| WPRO | 12.9 (0.0; 38.9) (10) | 39.6 (3.4; 86.1) (9) | 5.4 (0.0; 55.6) (11) | 51.4 (23.7; 91.7) (10) | 0.0 (0.0; 0.0) (2) | NA |
|  |  |  |  |  |  |  |
| **Income Level** |  |  |  |  |  |  |
| LIC | 15.8 (0.0; 98.0) (10) | 2.8 (0.0; 5.0) (7) | 27.3 (0.0; 100) (12) | 47.7 (0.0; 95.0) (10) | 15.0 (0.0; 30.0) (2) | 60 (60.0; 60.0) (1) |
| LMIC | 31.8 (0.0; 100) (25) | 22.5 (0.0; 92.7) (24) | 31.2 (0.0; 92.5) (24) | 46.6 (0.0; 100) (24) | 17.4 (0.0; 55.0) (13) | NA |
| UMIC | 29.7 (0.0; 89.0) (21) | 35.5 (0.0; 86.7) (20) | 29.4 (0.0; 80.0) (18) | 54.4 (10.8; 96.7) (21) | 26.4 (0.0; 65.0) (8) | 50.9 (11.9; 90.0) (2) |
| HIC | 25.8 (0.0; 100) (9) | 44.4 (0.0; 78.3) (8) | 33.4 (0.0; 96.7) (8) | 78.4 (0.0; 93.3) (9) | 53.3 (6.7; 100) (2) | 40.8 (0.0; 70.0) (3) |
|  |  |  |  |  |  |  |
| **Lowest price generic: public sector availability** | | | | | | |
| **All surveys** | 33.8 (0.0; 100) (70) | 25.9 (0.0; 100) (57) | 42.6 (0.0; 100) (64) | 42.3 (0.0; 100) (66) | 30.1 (0.0; 100) (28) | 28.5 (0.0; 59.1) (7) |
|  |  |  |  |  |  |  |
| **WHO REGION** |  |  |  |  |  |  |
| AFRO | 38.8 (0.0; 79.2) (18) | 12.0 (0.0; 62.5) (10) | 55.7 (0.0; 87.5) (16) | 44.5 (0.0; 88.7) (15) | 53.5 (0.0; 100) (6) | NA |
| EMRO | 40.4 (0.0; 100) (14) | 34.2 (0.0; 90) (13) | 46.8 (0.0; 100) (12) | 51.3 (0.0; 100) (14) | 6.7 (0.0; 43.3) (8) | 20.0 (0.0; 50) (3) |
| EURO | 43.6 (0.0; 100) (5) | 24.9 (0.0; 66.7) (3) | 24.7 (0.0; 62.9) (5) | 57.7 (0.0; 100) (5) | 25.0 (0.0; 50.0) (2) | 30.0 (30.0; 30.0) (1) |
| PAHO | 26.1 (1.9; 96.7) (11) | 22.2(0.0; 93.3) (11) | 33.1 (0.0; 84.6) (8) | 52.7 (4.5; 100) (10) | 12.4 (1.9; 33.3) (3) | 36.4 (16.7; 59.1) (3) |
| SEARO | 55.0 (6.0; 100) (9) | 34.0 (0.0; 85.0) (10) | 50.8 (0.0; 100) (10) | 47.0 (0.0; 100) (8) | 71.8 (0.0; 85.0) (6) | NA |
| WPRO | 27.0 (1.4; 70.0) (11) | 5.8 (0.0; 20.0) (8) | 25.1 (2.5; 94.4) (11) | 13.9 (0.0; 100) (10) | 35.0 (0.0; 70.0) (2) | NA |
|  |  |  |  |  |  |  |
| **Income Level** |  |  |  |  |  |  |
| LIC | 27.8 (0.0; 73.3) (14) | 17.0 (0.0; 78.6) (6) | 44.4 (0.0; 88.2) (12) | 9.3 (0.0; 83.3) (11) | 35.3 (0.0; 100) (3) | 0.0 (0.0; 0.0) (1) |
| LMIC | 39.8 (0.0; 100) (27) | 16.0 (0.0; 100) (26) | 50.9 (0.0; 100) (28) | 46.5 (0.0; 100) (26) | 30.2 (0.0; 100) (16) | 30.0 (30.0; 30.0) (1) |
| UMIC | 39.0 (0.0; 100) (21) | 32.2 (0.0; 93.3) (19) | 27.8 (0.0; 100) (18) | 40.6 (0.0; 100) (21) | 39.3 (0.0; 85.0) (8) | 13.4 (10.0; 16.7) (2) |
| HIC | 21.4 (0.0; 80) (8) | 40.7 (12.0; 70.0) (6) | 53.0 (0.0; 100) (6) | 25.0 (0.0; 86.7) (8) | 0.0 (0.0; 0.0) (1) | 47.5 (33.3; 59.1) (3) |
|  |  |  |  |  |  |  |
| **Lowest price generic: private sector availability** | | | | | | |
| **All surveys** | 46.8 (0.0; 100) (77) | 44.5 (0.0; 100) (66) | 42.2 (0.0; 100) (70) | 47.5 (0.0; 100) (70) | 22.0 (0.0; 83.3) (27) | 35.6 (0.0; 80.0) (7) |
|  |  |  |  |  |  |  |
| **WHO REGION** |  |  |  |  |  |  |
| AFRO | 52.3 (0.0; 100) (19) | 39.0 (0.0; 93.3) (13) | 44.7 (0.0; 100) (17) | 42.3 (0.0; 100) (17) | 25.2 (0.0; 48.3) (6) | NA |
| EMRO | 52.0 (0.0; 100) (15) | 61.1 (0.0; 100) (14) | 49.7 (0.0; 100) (14) | 50.7 (0.0; 100) (15) | 41.0 (0.0; 88.3) (8) | 68.9 (50.0; 80.0) (3) |
| EURO | 39.4 (2.9; 72.5) (7) | 27.1 (4.0; 81.5) (5) | 15.7 (0.0; 58.0) (6) | 51.7 (0.6; 83.3) (6) | 12.9 (0.0; 25.9) (2) | 12.0 (12.0; 12.0) (1) |
| PAHO | 50.4 (0.0; 93.2) (12) | 54.8 (14.3: 93.3) (11) | 44.3 (2.9; 94.1) (10) | 71.7 (0.0; 93.3) (10) | 4.6 (1; 9.6) (3) | 10.2 (0.0; 23.8) (3) |
| SEARO | 50.9 (11.4; 92.5) (11) | 58.6 (0.0; 95.0) (12) | 56.1 (14.3; 97.5) (10) | 57.3 (25.7; 92.5) (8) | 34.5 (0.0; 65.9) (6) | NA |
| WPRO | 34.8 (5.5; 86.1) (11) | 9.9 (0.0; 61.1) (9) | 18.0 (0.0; 88.9) (11) | 31.2 (0.0; 82.9) (12) | 0.0 (0.0; 0.0) (2) | NA |
|  |  |  |  |  |  |  |
| **Income Level** |  |  |  |  |  |  |
| LIC | 57.0 (0.0; 100) (14) | 48.0 (0.0; 98.2) (10) | 60.6 (0.0; 100) (14) | 55.2 (0.0; 100) (13) | 5.0 (0.0; 10) (2) | 80.0 (80.0; 80.0) (1) |
| LMIC | 39.9 (0.0; 95.0) (30) | 46.1 (0.0; 95.0) (28) | 42.4 (0.0; 100) (28) | 48.3 (0.0; 95.1) (29) | 28.9 (0.0; 83.3) (14) | 12.0 (12.0; 12.0) (1) |
| UMIC | 50.4 (5.5; 100) (23) | 41.8 (0.0; 100) (21) | 34.5 (0.0; 88.9) (20) | 45.3 (0.0; 100) (21) | 6.2 (12.5; 40.0) (9) | 41.7 (6.8; 76.7) (2) |
| HIC | 37.7 (0v; 100) (10) | 52.6 (0.0 ;93.3) (7) | 30.2 (0.0; 94.1) (8) | 48.5 (0.0; 80.0) (9) | 0.0 (0.0; 0.0) (2) | 24.6 (0.0; 50.0) (3) |
|  |  |  |  |  |  |  |

**Median price ratio of essential psychotropic medicines by WHO Region and country income level: lowest price generic**

|  | **Amitriptyline 25 mg cap tab** | **Fluoxetine 20 mg cap tab** | **Diazepam 5mg cap tab** |
| --- | --- | --- | --- |
|  | % (min; max) (n) | % (min; max) (n) | % (min; max) (n) |
|  |  |  |  |
| **Lowest price generic: public sector procurement price** | | | |
| **All surveys** | 1.76 (0.17; 6.18) (48) | 10.8 (0.33; 51.48) (27) | 3.28 (0.15; 37.41) (41) |
|  |  |  |  |
| **WHO REGION** |  |  |  |
| AFRO | 1.04 (0.17; 2.99) (12) | 11.06 (0.33; 39.07) (4) | 0.83 (0.19; 2.37) (14) |
| EMRO | 2.56 (0.82; 6.18) (12) | 9.13 (0.84; 45.98) (10) | 6.43 (0.23; 14.37) (8) |
| EURO | 2.96 (1.35; 5.13) (6) | 10.56 (3.46; 18.57) (3) | 3.47 (2.64; 4.27) (3) |
| PAHO | 1.96 (0.89; 4.61) (5) | 3.53 (2.22; 4.49) (3) | 1.34 (1.11; 1.58) (2) |
| SEARO | 0.85 (0.38; 1.76) (9) | 1.52 (0.38; 3.63) (4) | 0.40 (0.20; 0.73) (8) |
| WPRO | 1.53 (0.52; 2.34) (4) | 35.86 (24.07; 51.48) (3) | 9.20 (0.15; 37.41) (6) |
|  |  |  |  |
| **Income Level** |  |  |  |
| LIC | 1.28 (0.17; 2.99) (7) | 2.02 (0.33; 3.36) (3) | 1.01 (0.35; 2.72) (8) |
| LMIC | 1.78 (0.30; 6.18) (24) | 15.98 (0.38; 51.48) (11) | 3.49 (0.19; 37.41) (21) |
| UMIC | 1.91 (0.52; 4.61) (13) | 6.18 (1.12; 24.07) (7) | 1.43 (0.15; 4.40) (7) |
| HIC | 2.07 (0.51; 3.24) (4) | 11.08 (1.08; 32.03) (6) | 8.60 (0.70; 14.37) (5) |
|  |  |  |  |
| **Lowest price generic: public sector patient price** | | | |
| **All surveys** | 3.33 (0.0; 19.0) (43) | 25.18 (0.0; 350.76) (29) | 4.63 (0.0; 43.26) (36) |
|  |  |  |  |
| **WHO REGION** |  |  |  |
| AFRO | 4.18 (0.0; 19.0) (8) | 0.61 (0.0; 1.82) (3) | 4.05 (0; 11.46) (12) |
| EMRO | 1.19 (0.0; 5.26) (8) | 1.27 (0.0; 8.95) (9) | 1.95 (0.0; 6.97) (6) |
| EURO | 6.68 (3.12; 12.38) (4) | 35.57 (15.29; 55.85) (2) | 13.05 (10.57; 15.52) (2) |
| PAHO | 3.59 (0.0; 15.93) (8) | 0.0 (0.0; 0.0) (3) | 13.6 (0.0; 43.26) (5) |
| SEARO | 1.32 (0.0; 7.02) (9) | 1.25 (0.0; 8.08) (8) | 1.06 (0.0; 7.62) (8) |
| WPRO | 4.20 (0.0; 12.44) (6) | 158.93 (76.29; 350.76) (4) | 1.33 (0.0; 3.95) (3) |
|  |  |  |  |
| **Income Level** |  |  |  |
| LIC | 3.06 (0.0; 7.99) (5) | 3.59 (0.0; 8.95) (3) | 9.69 (0; 43.26) (7) |
| LMIC | 3.18 (0.0; 19) (21) | 35.16 (0.0; 350.76) (12) | 4.10 (0; 20.86) (19) |
| UMIC | 4.16 (0.0; 15.93) (13) | 28.01 (0.0; 107.24) (7) | 2.99 (0; 7.62) (7) |
| HIC | 1.81 (0.0; 7.23) (4) | 14.49 (0.0; 101.43) (7) | 0.0 (0.0; 0.0) (3) |
|  |  |  |  |
| **Lowest price generic: private sector patient price** | | | |
| **All surveys** | 15.97 (0.01; 85.08) (50) | 66.72 (0.01; 435.8) (43) | 20.76 (0.01; 82.1) (40) |
|  |  |  |  |
| **WHO REGION** |  |  |  |
| AFRO | 19.78 (0.01; 70.68) (11) | 87.16 (0.03; 397.96) (6) | 6.53 (0.01; 20.83) (11) |
| EMRO | 10.81 (1.97; 22.81) (7) | 73.80 (0.04; 326.96) (11) | 24.41 (9.31; 72.83) (7) |
| EURO | 6.36 (0.01; 11.57) (8) | 70.15 (0.01; 223.22) (4) | 27.35 (11.29; 52.77) (3) |
| PAHO | 32.65 (0.01; 85.08) (9) | 61.11 (0.01; 147.48) (9) | 42.21 (22.07; 82.1) (8) |
| SEARO | 10.72 (2.04; 16.72) (11) | 15.10 (3.80; 22.50) (9) | 17.92 (0.92; 27.73) (9) |
| WPRO | 10.7 (4.78; 17.17) (4) | 144.14 (9.74; 435.80) (4) | 3.28 (0.03; 6.53) (2) |
|  |  |  |  |
| **Income Level** |  |  |  |
| LIC | 12.28 (0.01; 33.17) (9) | 26.32 (0.03; 117.28) (6) | 16.64 (0.01; 52.77) (10) |
| LMIC | 16.6 (0.01; 85.08) (22) | 65.21 (0.01; 435.8) (19) | 20.82 (0.01; 82.1) (21) |
| UMIC | 15.6 (0.01; 46.54) (16) | 72.54 (0.01; 397.96) (12) | 25.81 (0.92; 72.83) (6) |
| HIC | 24.45 (17.69; 30.57) (3) | 100.24 (12.17; 326.96) (6) | 23.98 (6.91; 45.51) (3) |

**Affordability of essential psychotropic medicines by WHO Region and country income level**

| **Originator brand: public sector affordability** | | | | |
| --- | --- | --- | --- | --- |
|  | **Amitriptyline 25 mg cap tab** | **Fluoxetine 20 mg cap tab** | **Diazepam 5mg cap tab** | **Carbamazepine 200 mg cap tab** |
|  | % (min; max) (n) | % (min; max) (n) | % (min; max) (n) | % (min; max) (n) |
|  |  |  |  |  |
| **All surveys** | 0.7 (0.7; 0.7) (1) | 12.2 (10.1; 14.4) (3) | NA | NA |
|  |  |  |  |  |
| **WHO REGION** |  |  |  |  |
| AFRO | NA | NA | NA | NA |
| EMRO | 0.7 (0.7; 0.7) (1) | NA | NA | NA |
| EURO | NA | NA | NA | NA |
| PAHO | NA | NA | NA | NA |
| SEARO | NA | NA | NA | NA |
| WPRO | NA | 12.2 (10.1; 14.4) (3) | NA | NA |
|  |  |  |  |  |
| **Income Level** |  |  |  |  |
| LIC | 0.7 (0.7; 0.7) (1) | NA | NA | NA |
| LMIC | NA | 12.1 (12.1; 12.1) (1) | NA | NA |
| UMIC | NA | 10.1 (10.1; 10.1) (1) | NA | NA |
| HIC | NA | 14.4 (14.4; 14.4) (1) | NA | NA |
|  |  |  |  |  |
| **Originator brand: private sector affordability** | | | | |
| **All surveys** | 2.6 (0.3; 8) (34) | 13.8 (0.6; 58.7) (21) | 0.3 (0; 0.8) (8) | 2.8 (0.6; 6.8) (3) |
|  |  |  |  |  |
| **WHO REGION** |  |  |  |  |
| AFRO | 4.1 (2.2; 6.7) (5) | 43.2 (27.8; 58.7) (2) | 0.3 (0.2; 0.4) (3) | NA |
| EMRO | 1.5 (0.3; 3.5) (10) | 6.6 (3.0; 10.8) (5) | 0.1 (0; 0.2) (2) | 0.7 (0.6; 0.9) (2) |
| EURO | 3.3 (1.5; 4.9) (3) | 48.1 (48.1; 48.1) (1) | NA | NA |
| PAHO | 5.3 (0.9; 6.8) (6) | 14.2 (10.5; 15.8) (4) | 0.2 (0.2; 0.2) (2) | NA |
| SEARO | 1.3 (1.1; 1.9) (8) | 3.0 (0.6; 12.3) (5) | 0.5 (0.1; 0.8) (2) | 6.8 (6.8; 6.8) (1) |
| WPRO | 0.6 (0.5; 0.7) (2) | 12.9 (7.5; 16.4) (4) | NA | NA |
|  |  |  |  |  |
| **Income Level** |  |  |  |  |
| LIC | 3.7 (0.8; 6.7) (3) | NA | 0.3 (0.2; 0.4) (2) | NA |
| LMIC | 2.7 (1.1; 8.0) (15) | 15.1 (0.6; 58.7) (7) | 0.1 (0.1; 0.1) (1) | 6.8 (6.8; 6.8) (1) |
| UMIC | 2.5 (0.3; 7.2) (12) | 15.2 (3.5; 48.1) (9) | 0.3 (0.0; 0.8) (3) | 0.7 (0.6; 0.9) (2) |
| HIC | 1.8 (1.2; 2.8) (4) | 9.8 (3; 15.8) (5) | 0.2 (0.2; 0.2) (2) | NA |
|  |  |  |  |  |
| **Lowest price generic: public sector affordability** | | | | |
| **All surveys** | 0.9 (0.1; 2.6) (25) | 2.4 (0.1; 11.6) (10) | 0.1 (0.0; 0.2) (13) | 0.2 (0.2; 0.2) (1) |
|  |  |  |  |  |
| **WHO REGION** |  |  |  |  |
| AFRO | 1.1 (0.3; 2.6) (6) | 2.5 (2.5; 2.5) (1) | 0.1 (0.0; 0.1) (3) | NA |
| EMRO | 0.7 (0.1; 2.0) (4) | 0.3 (0.1; 0.4) (3) | 0.0 (0.0; 0.0) (2) | 0.2 (0.2; 0.2) (1) |
| EURO | 1.5 (0.3; 2.5) (4) | 0.9 (0.9; 0.9) (1) | 0.1 (0.1; 0.1) (1) | NA |
| PAHO | 1.0 (0.3; 1.4) (3) | 0.4 (0.1; 0.7) (2) | 0.1 (0.0; 0.2) (2) | NA |
| SEARO | 0.3 (0.3; 0.3) (2) | 0.2 (0.2; 0.2) (1) | 0.0 (0.0; 0.0) (1) | NA |
| WPRO | 0.7 (0.2; 1.8) (5) | 9.6 (7.5; 11.6) (2) | 0.1 (0.0; 0.2) (2) | NA |
|  |  |  |  |  |
| **Income Level** |  |  |  |  |
| LIC | 1.3 (0.3; 2.6) (6) | 1.4 (0.3; 2.5) (2) | 0.1 (0.0; 0.2) (5) | NA |
| LMIC | 1.3 (0.3; 2.5) (9) | 0.8 (0.7; 0.9) (2) | 0.1 (0.0; 0.2) (4) | NA |
| UMIC | 0.4 (0.1; 1.2) (9) | 4.0 (0.1; 11.6) (5) | 0.0 (0.0; 0.0) (3) | 0.2 (0.2; 0.2) (1) |
| HIC | 0.2 (0.2; 0.2) (1) | 0.1 (0.1; 0.1) (1) | 0.0 (0.0; 0.0) (1) | NA |
|  |  |  |  |  |
| **Lowest price generic: private sector affordability** | | | | |
| **All surveys** | 1.5 (0.1; 5.5) (54) | 3.6 (0.1; 26.6) (28) | 0.2 (0.0; 0.8) (27) | 1.6 (0.4; 4.6) (4) |
|  |  |  |  |  |
| **WHO REGION** |  |  |  |  |
| AFRO | 2.1 (0.5; 4.8) (12) | 3.9 (0.4; 10.1) (4) | 0.2 (0.0; 0.8) (7) | 4.6 (4.6; 4.6) (1) |
| EMRO | 1.1 (0.1; 2.7) (9) | 2.6 (0.1; 5.2) (5) | 0.1 (0.0; 0.2) (4) | 0.6 (0.4; 0.7) (2) |
| EURO | 1.9 (0.2; 4.7) (7) | 13.4 (0.8; 26.6) (3) | 0.3 (0.1; 0.7) (4) | NA |
| PAHO | 2.5 (0.5; 5.5) (9) | 2.1 (0.2; 3.6) (7) | 0.2 (0.0; 0.4) (6) | NA |
| SEARO | 0.8 (0.3; 1.1) (11) | 1.6 (0.4; 7.2) (6) | 0.0 (0.0; 0.1) (3) | 0.5 (0.5; 0.5) (1) |
| WPRO | 0.4 (0.2; 0.6) (5) | 4.4 (2.9; 6.0) (2) | 0.2 (0.2; 0.2) (1) | NA |
|  |  |  |  |  |
| **Income Level** |  |  |  |  |
| LIC | 2.2 (0.5; 4.8) (10) | 2.2 (0.4; 3.9) (4) | 0.2 (0.0; 0.7) (8) | 4.6 (4.6; 4.6) (1) |
| LMIC | 1.7 (0.2; 5.5) (25) | 3.1 (0.1; 12.8) (10) | 0.2 (0.0; 0.8) (13) | 0.5 (0.5; 0.5) (1) |
| UMIC | 0.8 (0.1; 3.0) (14) | 5.1 (0.1; 26.6) (9) | 0.1 (0.0; 0.2) (5) | 0.6 (0.4; 0.7) (2) |
| HIC | 1.1 (0.2; 2.6) (5) | 3.1 (0.2; 6.0) (5) | 0.0 (0.0; 0.0) (1) | NA |
|  |  |  |  |  |
